# Gravitational sliding of the Mt. Etna massif along a sloping basement

**DOI:** 10.1007/s00445-018-1209-1

**Published:** 2018-03-23

**Authors:** John B. Murray, Benjamin van Wyk de Vries, Andy Pitty, Phil Sargent, Luke Wooller

**Affiliations:** 1 School of Environment, Earth and Ecosystem Sciences, The Open University, Walton Hall, Milton Keynes, MK7 6AA UK; 2 Laboratoire Magmas et Volcans, Université Clermont Auvergne, Observatoire du Physique du Globe de Clermont, UMR6524-CNRS, Campus Universitaire des Cézeaux, 6 Avenue Blaise Pascal, TSA 60026 - CS 60026, 63178 Aubiere, Cédex France; 3 School of Architecture Design and the Built Environment, Department of Civil Engineering, Nottingham Trent University, 50 Shakespeare Street, Nottingham, NG1 4FQ UK

**Keywords:** Volcano deformation, Sector collapse, Analogue modelling, Hazard assessment, Volcano monitoring

## Abstract

Geological field evidence and laboratory modelling indicate that volcanoes constructed on slopes slide downhill. If this happens on an active volcano, then the movement will distort deformation data and thus potentially compromise interpretation. Our recent GPS measurements demonstrate that the entire edifice of Mt. Etna is sliding to the ESE, the overall direction of slope of its complex, rough sedimentary basement. We report methods of discriminating the sliding vector from other deformation processes and of measuring its velocity, which averaged 14 mm year^−1^ during four intervals between 2001 and 2012. Though sliding of one sector of a volcano due to flank instability is widespread and well-known, this is the first time basement sliding of an entire active volcano has been directly observed. This is important because the geological record shows that such sliding volcanoes are prone to devastating sector collapse on the downslope side, and whole volcano migration should be taken into account when assessing future collapse hazard. It is also important in eruption forecasting, as the sliding vector needs to be allowed for when interpreting deformation events that take place above the sliding basement within the superstructure of the active volcano, as might occur with dyke intrusion or inflation/deflation episodes.

**Electronic supplementary material:**

The online version of this article (10.1007/s00445-018-1209-1) contains supplementary material, which is available to authorized users.

## Introduction

Mt. Etna volcano dilates horizontally between its major flank eruptions. This outward expansion has been attributed to two processes: (1) gravitational spreading of its tall edifice and (2) inflation of a magma chamber within the volcano prior to eruption (Borgia et al. 2000; Lundgren et al. 2004; Neri et al. 2004; Obrizzo et al. 2004; Bonaccorso et al. 2006; Bonaccorso et al. 2013). However, it has long been noted that inter-eruptive horizontal displacement vectors measured from repeated GPS readings radiate not from the summit, but from an area WNW of it, leading to suggestions that the position of the inflating magma chamber is offset from the summit by up to a few kilometres, or that the magma chamber is tall and slopes in this direction (Nunnari & Puglisi 1994; Puglisi et al. 2004; Bonaccorso et al. 2011). This in turn implies that magma chamber inflation is the dominant process causing Etna’s dilation, since displacement vectors caused by gravitational spreading of a conical volcano should be radial to the summit of the edifice. Another peculiarity is the lack of symmetry in vector lengths, which are consistently much longer on the ESE side of the volcano. This has been interpreted with numerical and analytical models, in which the symmetrical radial movement of the inflating magma chamber is distorted by two large rectangular sub-horizontal dislocation planes dipping in different directions, that underlie the southern and eastern flanks in 1994–1995 (Bonforte and Puglisi 2003), or by a single larger dislocation plane beneath the lower eastern flank (Lundgren et al. 2003; Bonaccorso et al. 2006, 2013), or by a drag force due to subsidence of the continental margin on that side (Bonforte et al. 2011).

In the present paper, we present evidence that explains both the off-centre expansion and its asymmetry without recourse to these complex ad hoc models, and we also demonstrate, by the use of a simple Mogi model and a laboratory analogue gravitational spreading model, that our explanation applies in the case of the two abovementioned processes. The results of our work are applicable to other volcanoes of different types and in different situations.

## Field methods

**Fig. 1:**
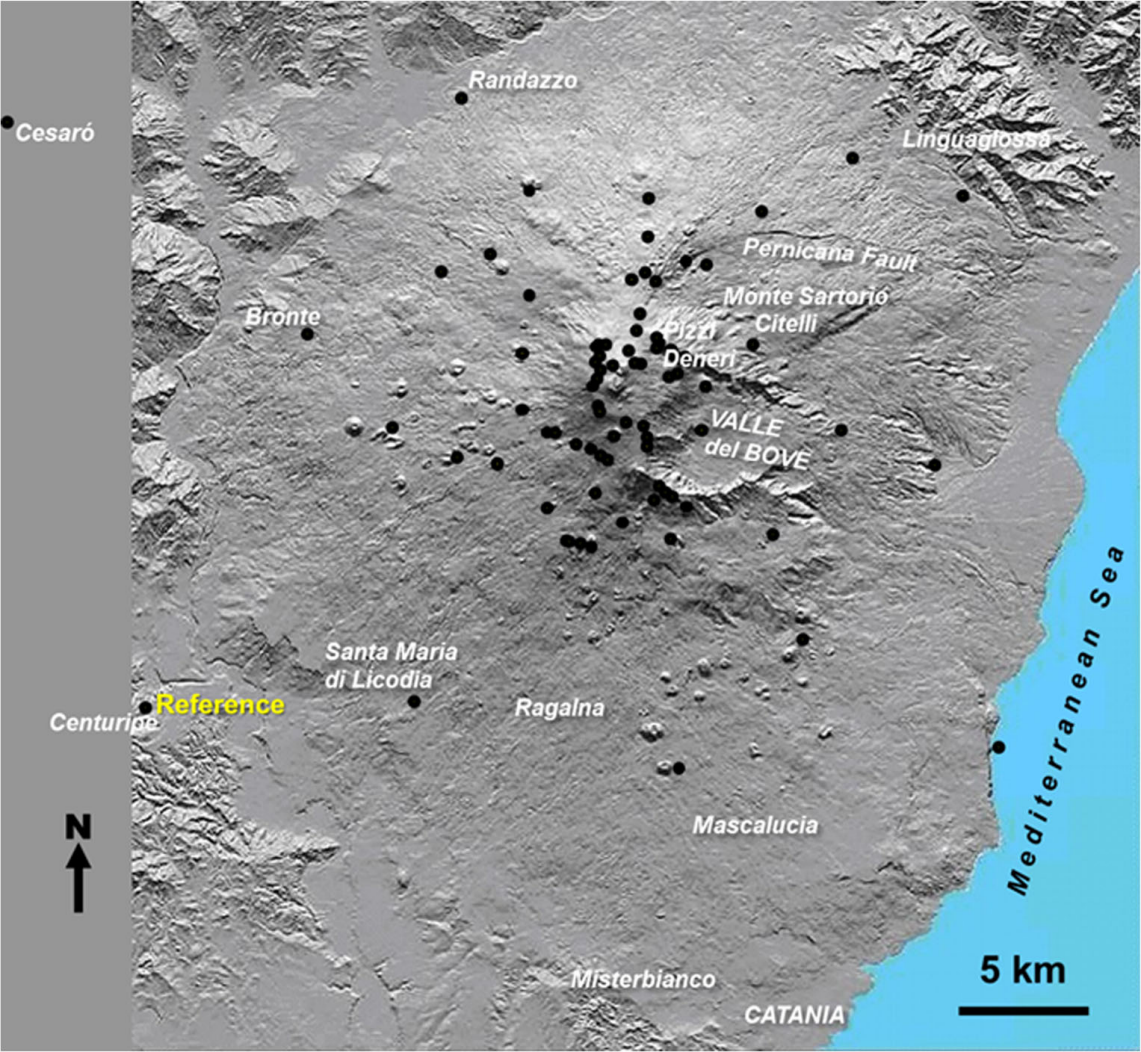
Map of Mt. Etna showing the network of GPS benchmarks (black circles) and the reference GPS point near Centuripe (left). The benchmarks shown are those extant in 2012. Also shown are towns, villages, and other locations mentioned in the text

A network that presently comprises over 100 benchmarks on Etna and surroundings (Fig. 1) was measured once a year with Leica system 530 GPS kits, with many lines observed on multiple days, to give mean error ellipses of major and minor axes 6.6 × 4.5 mm after full network adjustment (Reynolds 1934) using Leica GeoOffice software. Requests for the data should be addressed to the lead author. The times of surveys referred to in this paper are 2001 August, 2002 September, 2003 September, 2004 October, 2005 September, 2006 September, 2007 October, 2008 September, and 2012 September. It is a continual battle to replace benchmarks as they are destroyed by eruptive activity, and those in use currently are the remnant of a total of 200 benchmarks installed since 1981, half of which have been destroyed and successively replaced over the years, including 20 during the period covered by the present paper. The benchmarks are not the same as those later installed by the Istituto Nazionale di Geofisica e Vulcanologia (INGV) (Nunnari and Puglisi 1994), apart from the base stations near Centuripe and Cesaro, and seven other benchmarks that have been serendipitously found, so that the two networks can be tied into each other if necessary.

Regarding processing, some earlier workers (e.g., Puglisi et al. 2001; Bonforte et al. 2008) have tied their GPS networks into Continuously Operating Reference Stations (CORS) of the International Terrestrial Reference Frame (ITRF), between 100 and 550 km distant from Etna. They have then used three off-volcano reference stations around Etna instead of one reference station as we do. This is standard procedure for geodesists, who need to know positions relative to a universal reference frame, but in the present paper, we are concerned with the movement of the Etna volcano relative to its immediate surroundings. For the present analysis, the station near Centuripe, 4 km outside the southwest foot of the volcano and 24 km from the summit, is taken as stable and its coordinates held constant. This station was chosen because it rests on sedimentary rocks off the volcano, and outside the southeasterly mobile sector (Neri et al. 2004), but the choice of this reference station is not critical. If we take the mean of benchmarks near Bronte, Cesaro, Centuripe, Linguaglossa, Randazzo, and Santa Maria as stable (which together surround much of the volcano), the resulting mean error ellipse size of 4.5 × 2.5 mm does not differ significantly from that stated earlier; the differences are well within the measurement error.

Our method is less sophisticated than those of Puglisi et al. (2001) and Bonforte et al. (2008), but is sufficient to justify the conclusions of the paper, and there are some advantages to keeping it simple, in that no reference frame noise is introduced. We tested and compared the different methods using a larger external net and holding three stations fixed instead of one, but this procedure led to no substantial changes in the results. Further details of the comparisons are given in the supplementary material.

## Results

**Fig. 2:**
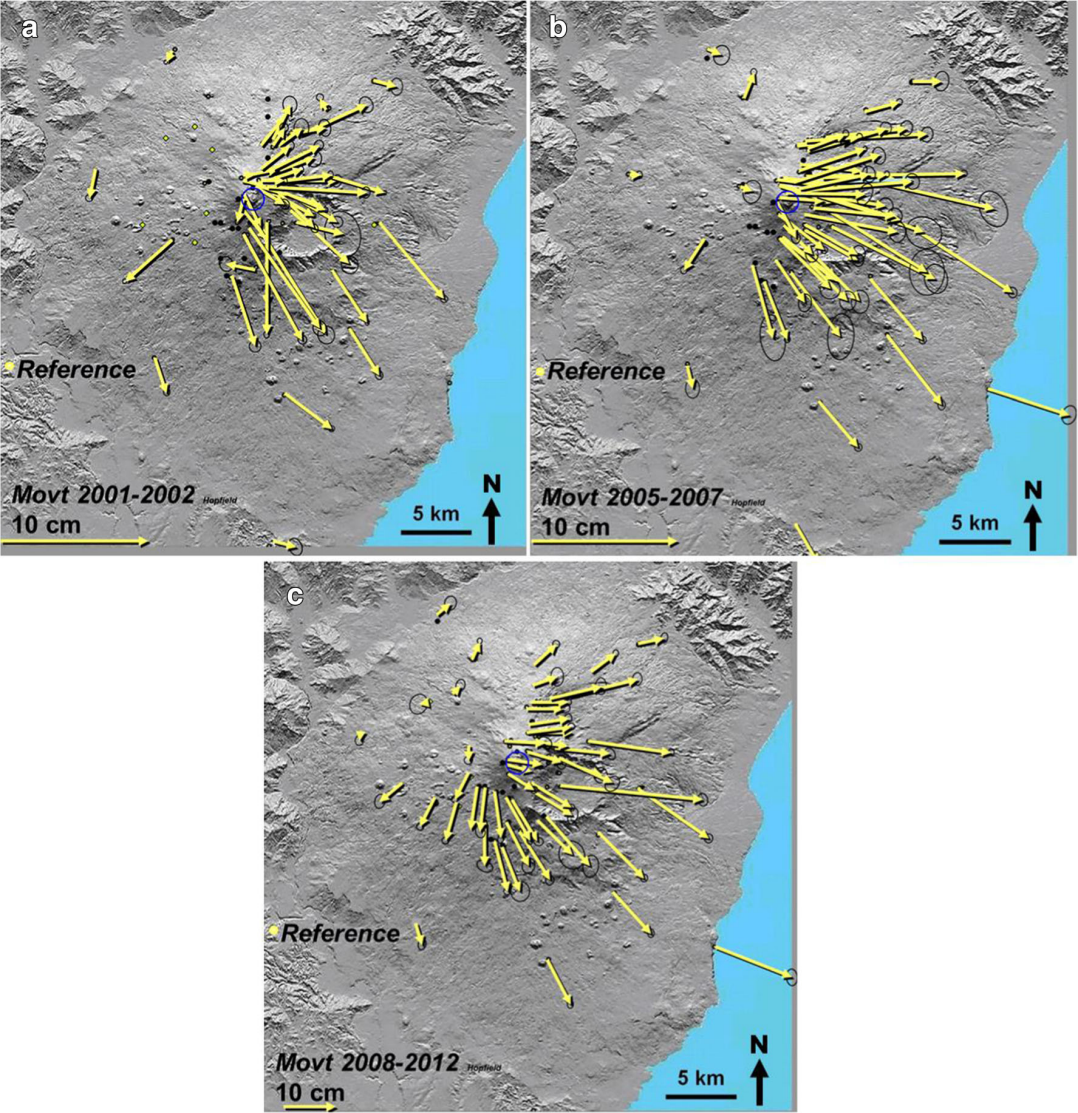
Maps of horizontal displacement vectors of GPS benchmarks on Mt. Etna between August 2001 and September 2002 (**a**), September 2005 and October 2007 (**b**), and September 2008 and September 2012 (**c**), measured between the three most recent major flank eruptions that began in 2001, 2002, and 2008. Vectors are relative to the reference station off the volcano, lower left. Note the different vector scale for map (**c**). Error ellipses are shown in black

Results of our measurements are presented in the horizontal displacement vector maps shown in Fig. 2a, b, and c, for the inter-eruptive periods August 2001–September 2002, September 2005–October 2007, and September 2008–September 2012. By “inter-eruptive,” we mean periods between the major flank eruptions that began in 2001, 2002, and 2008. All of these eruptions caused metre-scale displacements consequent upon dyke injection at the start of the eruption, which dominate the picture and hide the tiny movements dealt with here, which are one to two orders of magnitude smaller. The vectors in Fig. 2a,b, and c show that dilation occurs in each inter-eruptive period and that it is off-centre and asymmetric as described above.

**Fig. 3:**
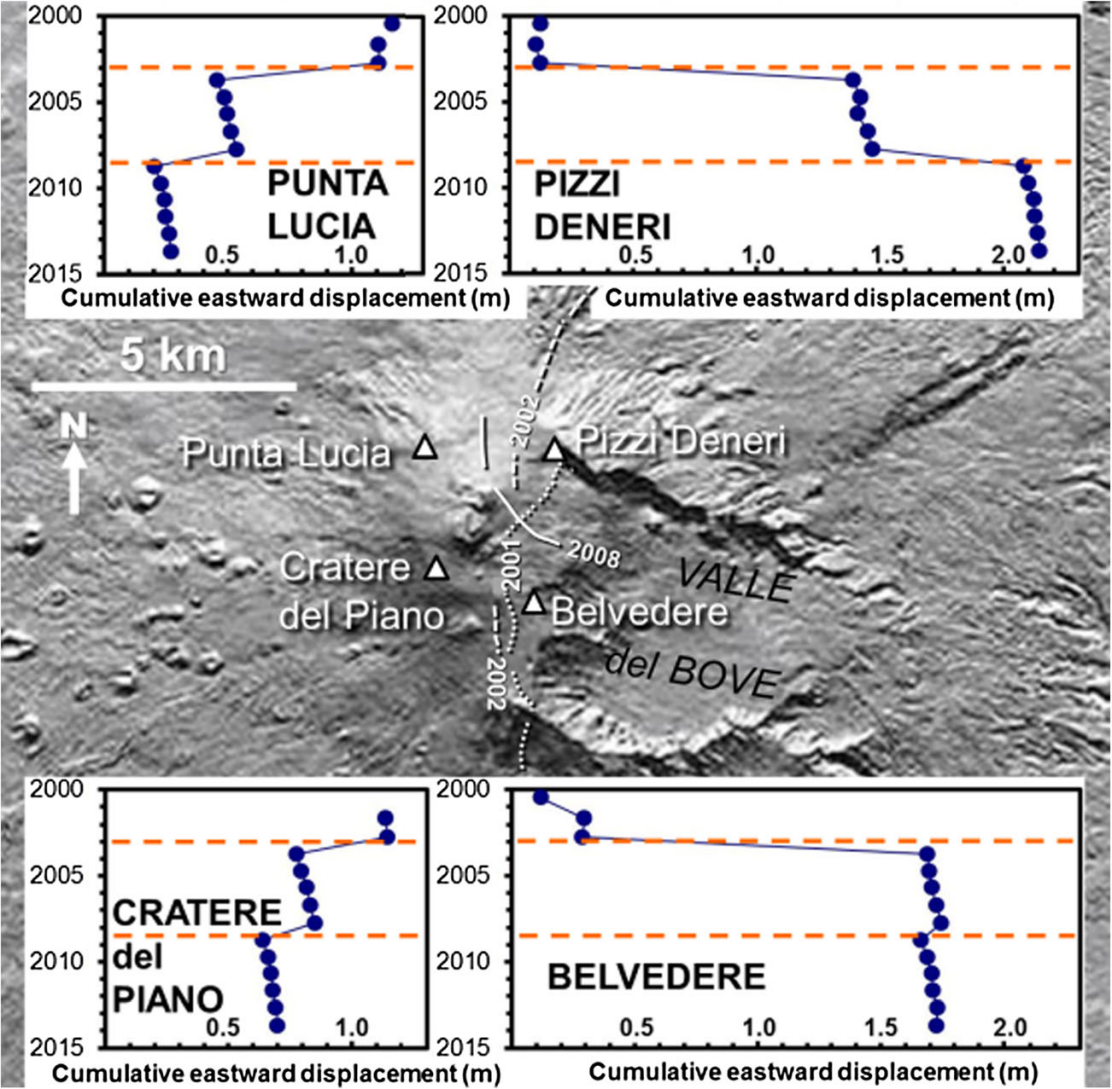
Plots of horizontal east-west displacement in metres (x-axis) at four locations surrounding the summit of Etna, against time (y-axis) from 2000 to 2012. Locations are shown in the central map, also positions of fissures associated with the three flank eruptions 2001 (dotted lines), 2002–2003 (dashed lines), and 2008–2009 (solid lines) that occurred during this period. The four graphs show that displacements of up to 1.4 m east and west occur during these eruptions (shown as dashed red lines in the graphs), but all stations drift back eastwards (downslope) in the time periods between them

The horizontal displacements that occurred during flank eruptions are illustrated by the series of graphs shown in Fig. 3.

These show the east-west horizontal displacements of four typical stations SW, NW, NE, and SE of the summit between 2000 and 2012. Although eastward movements of > 1 m have occurred during fissure opening at stations east of the summit, and westward movements of > 0.5 m at those to the west, the movement between these eruptions is consistently eastwards at a similar rate for all four stations. Movements at other stations confirm that this inter-eruptive downslope movement applies to the entire volcano.

## Distortion of displacement vectors at a dilating volcano by basement sliding

**Fig. 4:**
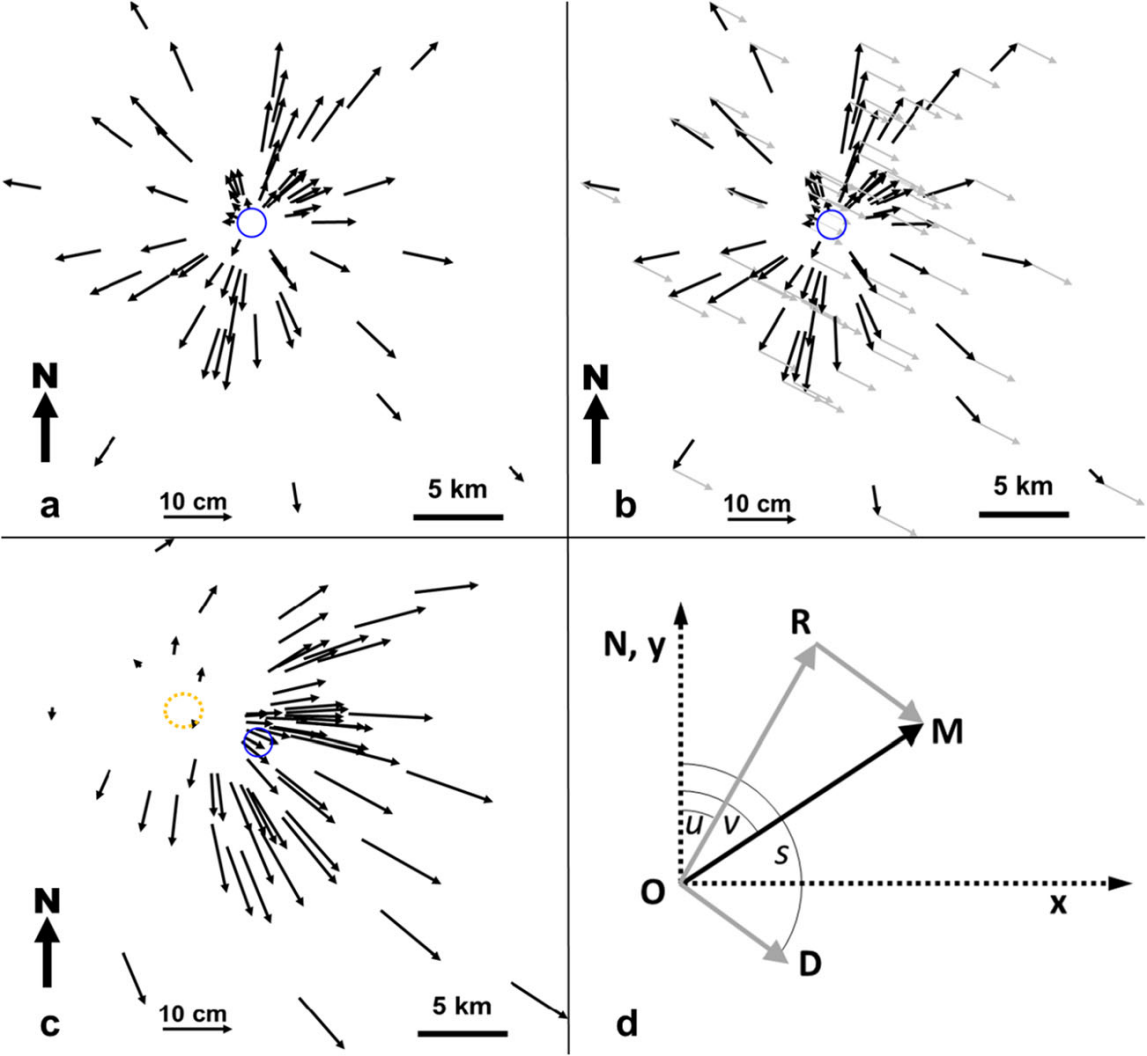
**a** Schematic of horizontal displacement vectors at Mt. Etna for a Mogi model point source at 10 km depth inflating 20 cm. The blue circle shows the position of the inflating source. **b** The same displacement vectors (black arrows) with sliding vectors of 6 cm, azimuth 120° (grey arrows) added to each vector, 120° being the approximate slope of Etna’s basement. **c** Horizontal displacement vectors resulting from the addition of inflating and sliding vectors. Note that vectors no longer appear to radiate from the inflation source (blue circle), but from an area WNW of it (orange-dotted circle), and are much longer towards the lower right, i.e., the direction of sliding. **d** Diagram illustrating the parameters used to derive the amount and direction of sliding from observed vector displacements. OR is the radial displacement vector, RM and OD the sliding vector, and OM the resultant observed vector of movement at a benchmark. Vector azimuths *u*, *s*, and *v* (radial, sliding, and observed, respectively) are also shown. The y axis is oriented north (*N*) and the x axis east. See text for details

A dilating volcano that expands perfectly radially will produce horizontal displacement vectors that are axisymmetric and radial to the centre of displacement (Fig. 4a). The length of these vectors will depend upon the cause of the expansion, which includes both magma chamber inflation and gravitational spreading. Both processes will effect an increase in vector length with distance from the summit up to a certain radius, after which the vector length will decrease.

In this paper, we are not concerned to distinguish the relative contribution of these two processes, but simply to investigate whether the entire volcanic edifice is sliding along its basement. In Fig. 4a, we show a map of vector lengths expected from a Mogi point source at 10 km depth that has raised the surface by 20 cm (radial black arrows). In Fig. 4b, a sliding vector of 6 cm with an azimuth of 120° (unidirectional grey arrows), roughly corresponding to the main direction of basement slope at Etna (Neri and Rossi 2002; Branca and Ferrara 2013), is added to each radial vector. In Fig. 4c, we show the vectors resulting from adding the sliding vector. The choice of 6 cm for the sliding vector is intended to be purely illustrative.

The addition of this vector has three important consequences. Firstly, vector lengths are shortened on the WNW side and lengthened on the ESE side. Secondly, vectors are no longer radial to the centre of displacement, but to locations WNW of it, largely within the orange-dotted circle shown in Fig. 4c. Thirdly, the vectors do not radiate from a single point, but from points increasingly further WNW of the summit, the further the vector origin is from the summit. This latter observation can be clearly seen in vectors in the top right and bottom left parts of Fig. 4c. A glance at the displacement vectors we have measured on Etna (Fig. 2), particularly between 2008 and 2012 (Fig. 2c), clearly shows similar characteristics.

## Deriving the sliding vector

To remove the sliding vector from the data in Fig. 2, and reveal deformation patterns resulting from other processes, it is first necessary to determine the direction and length of the sliding vector. For any volcano experiencing both symmetric radial expansion and unidirectional basement sliding, the amount of sliding for any observed interval can be derived as follows, illustrated in Fig. 4d. Note that azimuth is measured from North through East.

We define the following notation: *D* is the magnitude of the horizontal vector of downslope movement of the volcano (the sliding vector), *M* is the magnitude of the observed vector of movement at an individual station (the sliding plus radial vector), *s* is the azimuth of sliding in degrees, *u* is the azimuth of an individual station about the volcano summit in degrees, and *v* is the observed azimuth of displacement at an individual station in degrees. *O* is the origin of displacements, *N* is the north direction, OR the radial displacement, and OD the sliding displacement. RM is equal to OD (in direction and magnitude); OM is the resultant displacement. Angle NOR = *u* (assuming that the radial displacement is an expansion; if it is a contraction, *u* in this sense is 180° plus the azimuth of the station), angle NOM = *v*, and angle NOD = *s*.

The coordinates of *D* and *R* relative to *O* are

$$ D:\left(D\;\sin\;s,D\;\cos\;s\right) $$

$$ R:\left(R\;\sin\;u,R\;\cos\;u\right) $$

where *R* and *D* also double for the length of the displacements.

Therefore the coordinates of *M* are

1 $$ M\;\sin\;v=D\;\sin\;s+R\;\sin\;u $$

2 $$ M\;\cos\;v=D\;\cos\;s+R\;\cos\;u $$

Taking cos *u* times Eq. (1), sin *u* times Eq. (2), and subtracting:

3 $$ M\;\left(\sin\;v\;\cos\;u\hbox{--} \cos\;v\;\sin\;u\right)=D\;\left(\sin\;s\;\cos\;u\hbox{--} \cos\;s\;\sin\;u\right)\kern0.75em $$

Therefore,

4 $$ M\;\sin \kern0.37em \left(v\hbox{--} u\right)\kern0.37em =D\;\sin \kern0.37em \left(s\hbox{--} u\right), $$

hence,

5 $$ v=u+{\sin}^{-1}\;\left(D\;\sin\;\left(s-u\right)/M\right) $$

but care is needed to ensure the correct quadrant for *v*.

*M*, *u*, and *v* are observed values; *D* and *s* are varied iteratively to find the minimum sum of least squares of observed minus calculated values of *v* for all observed stations. Note that if the radial displacement OR exceeds the sliding displacement OD, stations on the western flank will continue to move westward, as is the case between 1994 and 2000 (Solaro et al. 2010; Bonforte et al. 2011).

Using the procedure described above, we derive the rate of horizontal sliding of the Etna massif for four inter-eruptive periods as in Table 1.

**Table 1:** Measured rates and directions of sliding

Inter-eruptive period	Magnitude of sliding displacement (*D* mm)	Rate of sliding (mm year^−1^)	Direction of sliding *s*
2001–2002	11 ± 7	10 ± 7	145° ± 27
2003–2004	17 ± 5	17 ± 5	117° ± 26
2005–2007	22 ± 9	11 ± 5	111° ± 33
2008–2012	61 ± 11	16 ± 3	120° ± 19
	Weighted means:	14 ± 4	120° ± 15

The variation in annual rate of movement is within the measurement error, so the sliding rate could be considered constant at about 14 mm per year. The graphs in Fig. 3 illustrate the similar rate of sliding at stations in different parts of the volcano throughout the period of observation, suggesting that the use of a constant rate for the sliding vector is justified.

**Fig. 5:**
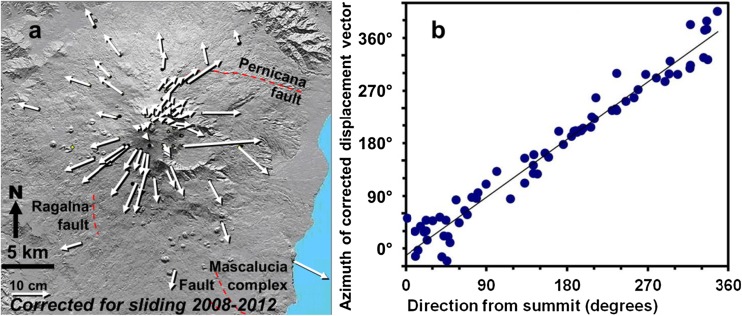
**a** map of corrected displacement vectors 2008–2012 with sliding vector subtracted. **b** Plot of azimuths of corrected displacement vectors for each benchmark against direction (North through East) of benchmark from the summit for the same period. Perfect radial movement would be a straight line. See text for details and discussion

Once the sliding vector has been derived for each point and subtracted from the observed displacement vector, the deformation without sliding can be portrayed and any other deviations from radial movement highlighted. This has been done in Fig. 5a for the period 2008–2012. This period was chosen because it is the longest (4 years long), so the vectors are correspondingly longer and the error ellipses correspondingly less important. It can be clearly seen that the vector lengths east and west of the volcano are much more balanced, and that most of the vectors radiate from the summit, rather than from an area WNW of it. Figure 5b shows how closely the vectors correspond to radial movement: a linear fit of the direction from the summit (angle *u*) against the azimuth of corrected displacement vector gives a coefficient of determination *r*^*2*^ of 0.94.

Looking at those vectors that deviate from radial, all of them are close to areas of local faulting (Borgia et al. 1992; Allard et al. 2006; Obrizzo et al. 2001; Walter et al. 2005; Alparone et al. 2012). Benchmarks near the Pernicana, Ragalna, and Mascalucia fault complexes (Fig. 5a) all deviate from radial by more than 20°. This is not surprising in the case of the Pernicana fault, which underwent acceleration in 2010, right in the middle of the 2008–2012 period (Guglielmino et al. 2011). The only exception is the station in the middle of the Valle del Bove. This is situated at the foot of a steep east-facing slope nearly 1 km high and has moved normal to the slope, with a corrected vector length about three times greater than other corrected vectors at similar distances from the summit.

Vertical movements attributable to the sliding should be uniformly downwards over the volcano by < 1 mm year^−1^, since the basement slope is so shallow. This is within the measurement uncertainty. Measured vertical movements during the above inter-eruptive periods show movements unrelated to the sliding: subsidence due to compaction of recent lavas around the summit (Murray 1988).

## Laboratory analogue modelling

**Fig. 6:**
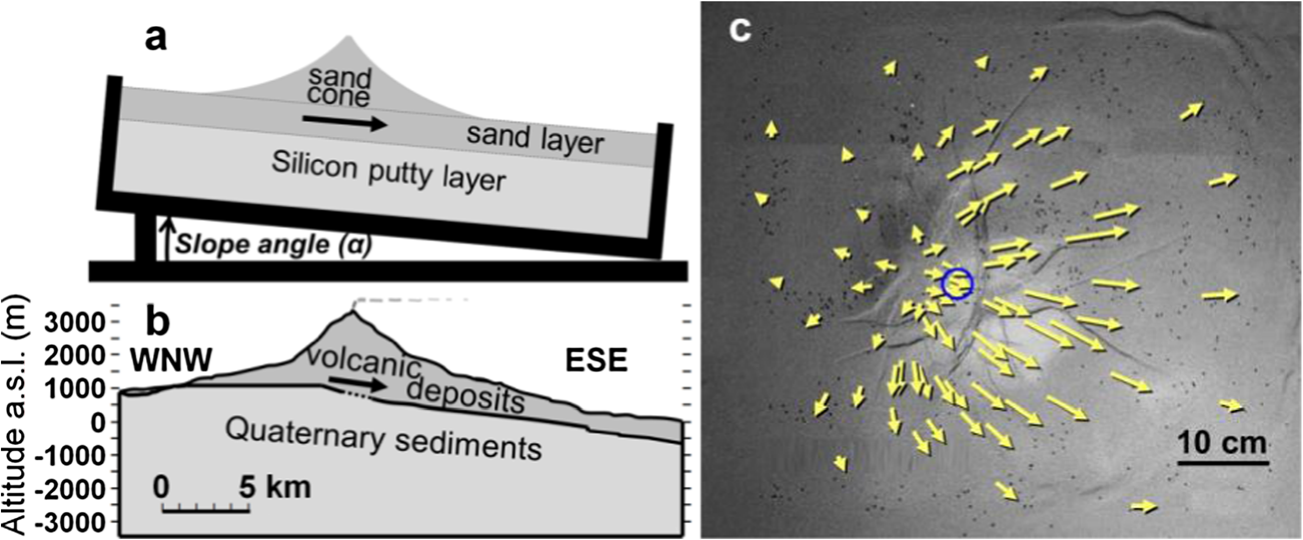
**a** Diagram of analogue model setup (Wooller et al. 2004). A flared cone of sand and plaster, representing a brittle volcano, overlies a sand and plaster layer representing a brittle fan of lavas. This in turn overlies a weak ductile silicon putty layer representing a sedimentary substrate on a rigid tilted base. **b** WNW-ESE section through Mt. Etna (2× vertical exaggeration) simplified from Branca and Ferrara (2013), showing the thickness of the volcanic cone and the lavas spreading out from its base, and the basement of ductile Quaternary sedimentary deposits with a sloping surface. The thick arrow in both diagrams shows the direction of measured movement. **c** Plan view of laboratory analogue model showing vectors of displacement after 15 min. The model is sloping 1° down to the right; the blue circle indicates the position of the summit of the sand “volcano.” Note the imbalance of vector lengths to the left and right, and the vectors radiating from positions upslope of the summit, also the fracturing and a rift-zone like structure (top)

We also looked at the effect of sliding at a volcano experiencing gravitational spreading alone, by re-examining the analogue model shown in Fig. 6 (Wooller et al. 2004). In this case, radial expansion is produced by the gravitational spreading of a sand and plaster cone on a weak ductile basement of silicon putty. The Mogi model used in the previous section assumes that Etna responds elastically, as do many of the models postulated by previous workers on this subject at volcanoes worldwide. An exception is Got et al. (2013), who found significant differences for surface deformation related to magma transport and dyking, when Piton de la Fournaise volcano is assumed to be elasto-plastic, rather than elastic. The sand and plaster cone in our analogue model also behaves elasto-plastically (Schellart 2000; Galland et al. 2015), and the silicon base is elasto-viscous (Delcamp et al. 2011), so differs from our previous Mogi model in assumptions about material behaviour. Figure 6a shows the setup of the analogue model (Wooller et al. 2004), and below it (Fig. 6b), a section through Mt. Etna showing the Etna cone and lavas and the weak and ductile Quaternary sediments beneath. The analogue model comprised a box with rigid base and sides containing a layer of ductile silicon putty, to represent the weak sedimentary basement beneath Etna. Overlying this is a brittle layer of mixed fine sand and plaster, corresponding to the apron of Etna lavas, and above this, a flared cone of sand and plaster representing the summit cone of Etna. The entire setup is tilted by 1°, to represent the effects of a sloping basement, and Fig. 6c shows the vectors of movement after 15 min. This model, further details of which are given in the Supplementary Material, shows radial expansion due to the volcano deforming under its own weight (i.e., gravitational spreading (Borgia 1994)) plus downslope sliding, which together produce apparent displacement of the expansion centre to the left of the summit, and longer displacement vectors on the downslope side, similar to the Etna inter-eruptive displacement vectors in Fig. 2. Because the slope of the Etna basement is roughly 120° azimuth, the Fig. 6c should be rotated clockwise by 30° to compare with the Etna results in Fig. 2.

## Discussion

Our results are broadly similar to GPS data published by other workers (Bonaccorso et al. 2006, 2011; Bonforte et al. 2008), bearing in mind that slightly different time periods are involved, and different base stations used. They are also similar to measurements of deformation in the period 1993–2000 between the major flank eruptions of 1991–1993 and 2001 (Bonaccorso et al. 2011; Bonforte et al. 2011). In every case, there is strong asymmetry in displacement vector magnitudes, much longer deformation vectors occurring to the ESE, and the vectors do not radiate from the summit, but from points to the WNW of it. InSAR is a useful technique that complements GPS measurements, but InSAR data are blind to the north-south component of movement, due to satellite configurations, so cannot be used to measure displacement vector length nor direction, but only the east-west component of movement. Solaro et al. (2010) have a table of east-west displacements which covers the period 2003–2008, taking a station in Catania as stable, though actually this is in the unstable SE sector of Etna and might be subject to local fault movement. Nonetheless, it is clear from their data that east flank stations show persistently eastward movement between 2003 and 2008, whereas the west flank stations show generally smaller movements with no overall trend eastwards between major flank eruptions.

**Fig. 7:**
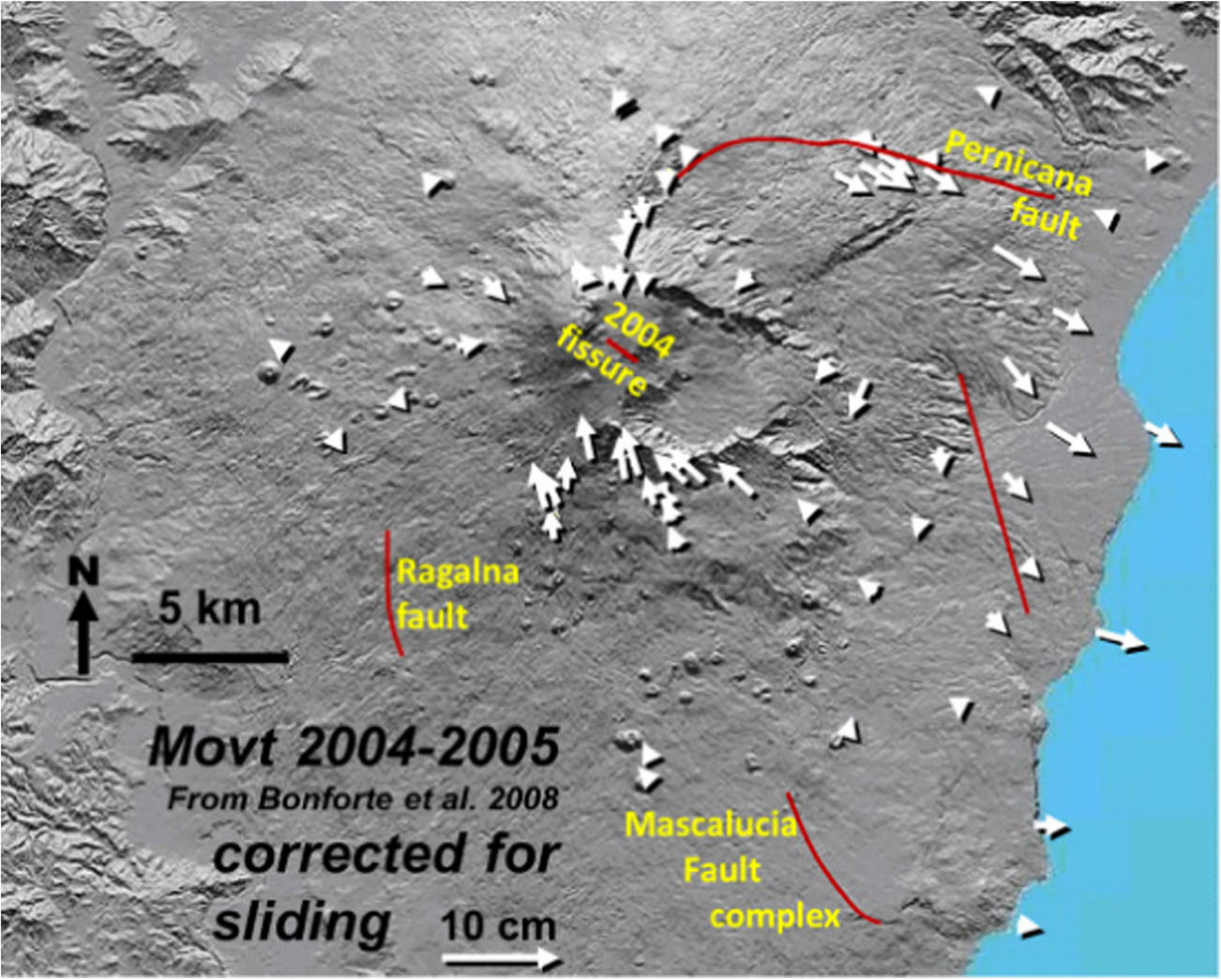
Map of horizontal displacement vectors between July 2004 and July 2005, showing not the measured displacement, but displacements after the sliding vector has been subtracted from each individual displacement vector as in Fig. 5a. The main fault complexes are shown in red. All data derived from values of horizontal deformation published in Bonforte et al. (2008), as our stations were not occupied at these times. Subtracting the sliding simplifies and clarifies the volcanic situation, providing a picture of contraction around the summit and 2004 fissure and left lateral displacement of the Pernicana fault. See text for further details

It would be interesting to know what happens during periods of horizontal contraction instead of expansion: does Etna continue to slide during periods of deflation? Unfortunately there are no periods of contraction in any of the inter-eruptive periods we are considering. Some episodes of horizontal contraction have occurred outside these inter-eruptive periods, e.g., during the period July 2004 to July 2005, interpreted by Bonforte et al. (2008) as a deflation associated with the 2004 eruption that occurred during this interval. The 2004 eruption was noteworthy for the generally small amounts of deformation, behaving like a summit eruption in that respect, so can be regarded as similar to an inter-eruptive period. If we subtract the sliding vector from Bonforte et al.’s (2008) published data (Fig. 7), it is clear that horizontal contraction has indeed occurred all around the summit, with vectors pointing towards the summit, the eruption site, and the area of subsidence on the north flank. Like the period 2008–2012, also seen is clear displacement associated with the well-known areas of local faulting, particularly the Pernicana fault, where a small left-lateral displacement averaging 4 cm has taken place, though this movement diminishes with distance from the fault.

Dozens of short explosive summit eruptions occurred in the period 2011–2012, when a new summit cone adjacent to the Southeast Crater was formed (Behncke et al. 2014). Deformation related to magma movements associated with these eruptions included periods of contraction, notably in 2011, but this was small: of the order of 5 ppm (Patané et al. 2013) and it has not affected the overall dilation between 2008 and 2012 (Fig. 2c).

Regarding the relationship between eruptive fracturing and basement sliding, geophysical measurements confirm that intrusions take place within the volcanic edifice (Sanderson 1982; Murray and Pullen 1984; Bonaccorso 1996; Bonaccorso et al. 2002; Aloisi et al. 2003, 2009) whereas the uniformity and direction of the basement sliding are consistent with it taking place in the ductile sediments below the base of the volcanic pile. However, it is likely that the basement sliding will have an influence, for example on the position and orientation of the eruptive fractures, due to changes in the stress field at the volcano/basement interface, brought about by sliding.

The sloping model basement used in the laboratory analogue model is planar, in contrast to the Etna basement surface which is an almost horizontal plateau surface beneath the NW flanks, with a 17-km wide horseshoe-shaped depression scooped out of the eastern and southeastern sector (Neri and Rossi 2002; Norini and Acocella 2011; Branca and Ferrara 2013), that reaches a depth of about 400 m, and includes the summit craters, which lie about 1 km inside its northeastern edge. The overall slope of the basement is towards the ESE, and Fig. 6b shows a WNW-ESE section through the volcano showing the basement topography, compared to the model planar substratum (Fig. 6a). However, the present basement of Etna is the end product of tens of thousands of years of gravitational spreading (Borgia et al. 1992), and of conduit formation below the summit. At the end of our laboratory experiment, the silicon putty basement also had a pronounced valley downslope from the summit, caused by the basement sagging under the weight of the sand volcano, as is the case with all such experiments (Merle and Borgia 1996; Acocella et al. 2013b). The effect of a different starting basement slope angle is demonstrated in Wooller et al. (2004). On Etna, the sedimentary basement outcrops near Vena, 700 m above sea level on the NE flank, and the Pernicana Fault displaces both the sedimentary basement and the volcanic superstructure (Neri et al. 2004), so it is highly likely that the basement is involved in the sliding, which will have modified its original surface.

Regarding the cause of the component of radial expansion between eruptions, we have used two different models. One simulates a magmatic inflation on a sliding basement (Fig. 4) in which the component of radial expansion is the direct result of magma pressure, and the other gravitational spreading on a shallow slope (Fig. 6) in which radial expansion is caused by the volcano deforming under its own weight (Borgia et al. 1992; Borgia 1994). Both models reproduce the main features of Etna’s horizontal deformation; it remains to be seen which of the two processes is dominant. In this paper, we have only considered horizontal movements; adding the vertical component of movements could help discriminate the two models. Our model demonstrates that if inflation of a magma chamber is causing the dilation of Etna, then the chamber is directly beneath the summit, rather than WNW of it as suggested by previous authors (Nunnari & Puglisi 1994; Puglisi et al. 2004; Bonaccorso et al. 2011).

This is the first time that current persistent basement sliding of the entire edifice has been detected and measured on an active volcano. These results are important because there is strong geological evidence that volcanoes that have slid downslope in this manner have a propensity to experience very large catastrophic sector collapse on the downslope side later in their history, as at Socompa, Chile (Wooller et al. 2004), and at Colima, Mexico, which is situated on the steep slopes of the larger, older Nevado da Colima (Cortés et al. 2010). Such events are rare, occurring about four times a century worldwide (Siebert 1992), but have devastating consequences, so the possibility of future occurrences needs to be taken seriously, both at Etna and other volcanoes. It has already been suggested that a sloping substrate may be presently playing a major role at two other volcanoes. Teide volcano, Tenerife, is built upon a 5° sloping clay-rich substratum and has evidence of downslope movement (Marquez et al. 2009), and slow sliding or spreading is taking place at Piton des Neiges volcano, which may have been a contributory factor to the large flank collapse events at Piton de la Fournaise volcano (Le Friant et al. 2011), built upon its flanks (Upton and Wadsworth 1965). Colima volcano is also showing signs of downslope movement (Murray 1993; Murray and Wooller 2002), which may result in another sector collapse, which has already occurred at least five times on this volcano (Cortès et al. 2010).

Regarding possible future major slope failure at Etna, this has been discussed many times following an episode in the 1980s when a 2-km sector of the upper eastern flank of Etna began subsiding at an accelerating rate, some levelling stations attaining nearly 2 m of subsidence before stabilizing. Tilting of stations at Pizzi Deneri and Citelli between 1980 and 1987 suggested that much of the NE flank of the volcano was affected (Murray et al. 1994). There were small local slope failures during east flank eruptions in 1986, but the feared catastrophic sector collapse did not occur. These events inspired the E.U.-funded multi-disciplinary EPOCH project 1990–1993 on slope stability at Etna. More recently, the Italian national FLANK project, also on flank instability at Etna (Acocella et al. 2013a), has revived interest in slope failure. The EPOCH project emphasized the importance of gravitational spreading and slope metastability/intrusion interaction (Borgia et al. 1992; Murray et al. 1994) on potential slope failure, whereas the FLANK project looked at a wide range of factors such as degassing (Federico et al. 2011), faulting (Bonaccorso et al. 2013), intrusions and extensional tectonics (Bonaccorso et al. 2011; Norini and Acocella (2011). Poland et al. (2017), summarizing knowledge of volcano instability worldwide, and particularly at Etna, Kilauea, and Piton de la Fournaise, propose two principal driving forces: gravitational spreading, which dominates at Kilauea, and magmatic activity, which dominates at Etna and Piton de la Fournaise.

We would suggest that basement gravitational sliding is a third force, though it is as yet unclear what part this plays in relation to events and mechanisms discussed in the previous paragraph. The sliding identified in this paper is a slow precursive phenomenon, likely to be important over very long time scales, which may eventually prime the edifice for a major collapse and confine it to one preferential direction. Such large edifice-wide events may include large amounts of substrata, like at the Heart Mountain slide, Wyoming (Anders et al. 2011).

Such an event is likely to be triggered by a large, brief event of an intrusive or seismic nature (Bonaccorso et al. 2013), perhaps aided by hydrothermal weakening, for which there is some evidence at the present time (Behncke et al. 2008; Liotta et al. 2010). Such a scenario has also been invoked for the Heart Mountain slide (Mitchell et al. 2015).

However, the low velocity of sliding, amounting to 1.4 m per century at present, is at least four orders of magnitude lower than that observed prior to the Mount St Helens event (Voight et al. 1983). Failure is likely to be preceded in the short term by a progressive acceleration in downslope movement (Voight and Cornelius 1991; Murray et al. 1994). This could be missed if data are interpreted without allowing for the effect presented here.

## Conclusions

We conclude that entire edifice of Mt. Etna is sliding downslope towards the Mediterranean Sea at an average rate of 14 mm per year. The sliding is lubricated by the weak sediments beneath Etna and effected by the slope of the basement. We propose that the complex depression in the basement/volcano interface beneath the ESE flanks of the volcano (Fig. 6b) is the result of the gradual sinking of the volcanic pile into the weaker basement.

The sliding is independent of the fracturing of the volcano that occurs during large flank eruptions, which takes place within the volcanic edifice, though sliding may play a key role in the positioning and the nature of eruptive fracturing.

It is possible that the observed downslope sliding may eventually lead to greater risk of large scale slope failure, though there is no sign of this happening at the present time.

Similar sliding may be taking place at other active volcanoes on sloping basements, such as Colima (Mexico), Teide (Tenerife), and Piton des Neiges (Reunion Island), all of which show features consistent with it.

Finally, our results indicate that basement sliding can seriously compromise the interpretation of deformation data. In future, the sliding vector should be removed from displacement vectors at Etna and similarly affected volcanoes using the procedure described under “Deriving the sliding vector” above, both to clarify surface deformation patterns and to monitor any changes in the rate of downslope movement for hazard assessment.

## Electronic supplementary material

ESM 1 (DOCX 72 kb)
