# Supplementary material for: Gravitational sliding of the Mt. Etna massif along a sloping basement
Source: Bull Volcanol. 2018 Mar 23;80(4):40. doi: 10.1007/s00445-018-1209-1 (PMC6560784; doi:10.1007/s00445-018-1209-1)
Supplement: Supplementary file 1 — (DOCX 72 kb) [file 445_2018_1209_MOESM1_ESM.docx]

Supplementary material for

Gravitational sliding of the Mt Etna massif along a sloping basement

John B. Murray^1^*, Ben van Wyk de Vries^2^, Andy Pitty^1^, Phil Sargent^3^, Luke Wooller^1^.

correspondence to: [j.b.murray@open.ac.uk](mailto:j.b.murray@open.ac.uk)

The reference frame of Mt Etna:

Some earlier workers (e.g. Puglisi et al 2001, Bonforte et al 2008), have tied their GPS networks into Continuously Operating Reference Stations (CORS) of the International Terrestrial Reference Frame (ITRF). They have also used 3 off-volcano reference stations around Etna instead of one reference station as we do. This procedure is standard for geodesists, who need to know positions relative to a universal reference frame, but in the present paper we are concerned with the movement of the Etna volcano relative to its immediate surroundings. To test the difference between the two approaches, we used the above method for the period 2008-2012, to see what difference it made to the horizontal displacement vectors on the map shown in fig. 2(C). We tied our network into 3 CORS stations at Noto (100 km south of Etna), Matera (on the mainland of Italy, 350 km NNE), and Cagliari on the island of Sardinia, 550 km WNW of Etna. We then processed the data for the two epochs holding 3 stations near Cesaro, Bronte and Centuripe fixed. The resulting vectors differed in azimuth by a mean of 2°.4, and the maximum difference in the position of the end of the vector was 13 mm, or 3mm per year. This is insignificant when compared to the map in fig. 2(C), translating to a 1mm difference on the page when printed at A4 size.

Palano et al. (2010) have discussed the effect of non-volcanic movements of stations both near to and far from Etna, and how these may cause apparent displacements and rotation of the GPS network. They have proposed a geodetic reference frame based on the movements of 3 networks, with stations up to 350 km distant, during the period 1996 to 2009. However, we were using a different set of benchmarks, based around an earlier summit network (Murray & Pullen 1994), and also have to face the practicalities of stations that are lost under pyroclastics or lava, or destroyed by public works, or not measured for other reasons. Therefore, like other workers, we have referenced this to a single station that has not moved in relation to other similar stations. In our case we have used the station near Centuripe, which lies in sedimentary rocks off the volcano, well outside the mobile sector found by Neri et al (2004). Regarding other possible base stations outside the mobile sector at Cesaro, Bronte and Randazzo, use of these would have made little difference to the overall results. However, in no case do the independent movements of these possible reference stations show any sign of rotation in any of the period considered.

**Further details of the laboratory analogue model:**

The experimental setup is shown in Fig. S1. A flared cone of sand and plaster, representing a brittle volcano, overlies a sand and plaster layer of thickness *Hb* representing a brittle substrate. This in turn overlies a weak ductile silicon putty layer of thickness *Hd* on a rigid tilted base. The brittle cone and the brittle layer beneath it are shaded dark grey, and the weak ductile layer light grey. Both brittle materials are dry, sieved Fontainebleau sand containing 13% plaster. Rigid boundaries are used to avoid elastic responses to the model weight (Wooller et al 2004).


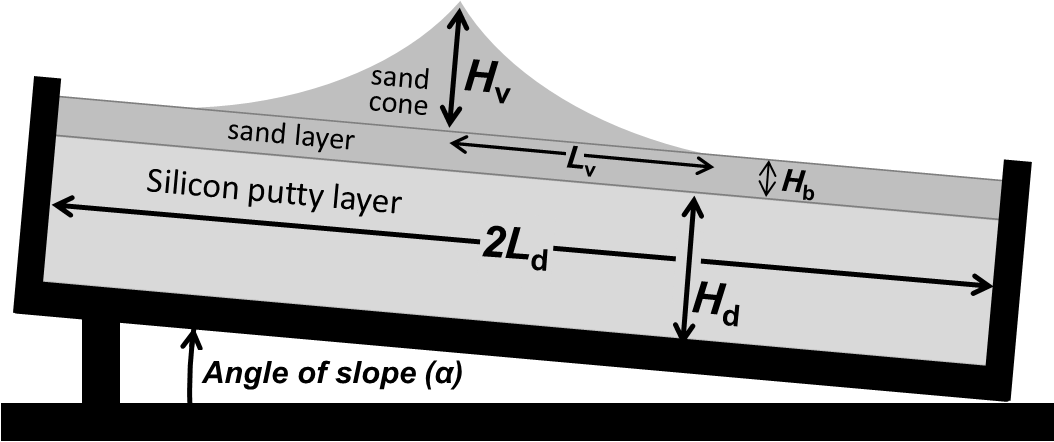


**Fig. S1.** Diagrammatic cross section of experimental setup, showing parameters referred to in the text and listed in table 1.

**Scaling analysis:**

The scaling analysis is based on the Buckingham-Π theorem, and uses information on selected geometric variables and material properties listed in Table S1. Gravity (*g*) is the only driving force, and the time-span of deformation is T. A scaling method similar to that in (Borgia et al (2000a)) is used, but with an extra term *Π10* to describe the substrate dip (Wooller et al 2004). The Buckingham-Π theorem states that for this system there are 13 variables, which, minus three dimensions (space, mass and time), leaves 10 dimensionless parameters. The first five of these are the geometrical ratios of the system (Table S2, *Π1* to *Π5*). Next, there are two ratios between the major pressures of the system (Table S2, *Π6* & *Π7*). This is a measure of the buoyancy of the substratum, and determines both the faulting density within the volcano and the rate of spreading. Following on from this, the next two dimensionless parameters (Table S2, *Π8* & *Π9*) are ratios between the gravitational, viscous and inertial forces. *Π9* defines the rate and time-span of deformation. Finally, there is the slope angle of the underlying weak substrata, *Π10*. Slope in the model, being a ratio of two lengths, is equal to that in nature. Using the Table S1 values, 10 dimensionless numbers are derived for Mt Etna and the model, and listed in Table S2 below, values of *T* for Etna being adjusted to the vector scales in Fig. 2. The values for Etna and the model largely correspond, considering the uncertainties of some of the Etna parameters.

References:

Borgia A, Delaney PT. Denlinger RP, 2000a, Spreading volcanoes: Ann.Rev. Earth. Planet. Sci. 28: 539–570.

Table 1.

Values for geometric variables and material properties on Etna, and in the laboratory model

_______________________________________________________________________

Variable______________________Units_____Etna________Model______Nature/Lab._

*Hv* Volcano height m 2500 0.05 5 x 10^4^

*Lv* Volcano radius m 2600-12000 0.08 10^4^ - 10^5^

*Le* Volcano geometric radius m 8200 0.063 1.3 x 105

*Hb* Brittle substrata thickness m 400-1000* 0.01 ~4 - 10 x 10^4^

*Hd* Ductile substrata thickness m 2000-4000* 0.01 ~3 x 10^5^

*Ld* Substratum radius m 3 x 10^4^* 0.75 10^4^ – 10^5^

*α*  Substratum dip ° <1°- 3° 1° ~0.5 - 3

*ρv* Volcano cone density kg m^-3^ 2000-2500 1350 ~1.7

*ρb* Brittle substrata density kg m^-3^ 2000-2500 1350 ~1.7

*ρd* Ductile substrata density kg m^-3^ 1800-2000 1000 ~1.9

*Φ* Angle of internal friction ° 30-40° 33° 0.9 – 1.4

*μd* Viscosity of ductile layer Pa s 1 x 10^17-21^ 3 x 10^5^ 10^12^ – 10^16^

*g* Gravity m s^-1^ 9.81 9.81 1

*T* Time-span of deformation s 3-12 x 10^7^ 10,000 10^3^ – 10^4^

Values for *Hb* & *Hd* are from Branca & Ferrara (2013). *Values with an asterix are approximate.

Table 2.

Dimensionless numbers used in scaling process

_______________________________________________________________________

Description___________________________________Etna________________Model________

*Π1*  Potential instability  *Hv* / *Lv* 0.2 - 1 0.6

*Π2* Strength of substratum *Hb* / *Hv* 0.2 - 0.4 0.2

*Π3* Weakness of substratum *Hd* / *Lv* 0.3 - 0.8 0.1

*Π4* Scaled volcano radius *Lv* / *Le* 0.3 - 1 1

*Π5* Scaled system radius *Ld* / *Lv* 4 12

*Π6* Volcano floating potential *ρdHd* / *ρvHv* 0.7 – 1.3 0.15

*Π7* Brittle substrata floating potential *ρdHd* / *ρbHb* 3 – 5 1

*Π8*  Process restraint 2*Nϕ Hb*/*Hd* *Hb* /*Hv ρb* /*ρv* 0.1 – 0.6 0.01

*Π9*  Process rate(3*cμ*/*ρvgHvT*) (*Lv*/*Hv*) 0.1 - 57 35

*Π10* Substrata slope Atan(*Ht*/*Ld*) ~0°.2 - ~2°.8 1°

______________________________________________________________________
